# Detección de ADN del gen *ompA* de *Chlamydia trachomatis* en orina mediante amplificación isotérmica mediada por bucle (LAMP)

**DOI:** 10.1515/almed-2025-0061

**Published:** 2025-05-29

**Authors:** Hemali Attanayake, Charitha Goonasekara, Nalaka Abeygunasekera, Jayanthi Elvitigala, Kamani Mangalika Gunasekera

**Affiliations:** National STD AIDS Programa de Control, Colombo, Sri Lanka; Departamento de Ciencias Pre-clínicas, Facultad de Medicina, Universidad General Sir John Kotelawala Defence, Ratmalana, Sri Lanka; STD Clinic, Hospital Colombo South Teaching, Kalubowila, Sri Lanka; Departmento de Microbiología, Facultad de Ciencias Médicas, 92953 Universidad de Sri Jayewardenepura, Nugegoda, Sri Lanka

**Keywords:** *Chlamydia trachomatis*, extracto crudo, amplificación isotérmica mediada por bucle (LAMP), amplificación isotérmica mediada por bucle, orina

## Abstract

**Objetivos:**

Actualmente, existen en el mercado kits de PCR en tiempo real eficaces para la detección de material genético de *Chlamydia trachomatis* (CT), aunque estos tienen un elevado coste. En los países con recursos limitados, resulta esencial disponer de pruebas diagnósticas económicas, con una buena sensibilidad y especifidad, para la detección de infección por *C. trachomatis.* El objetivo de este estudio es describir la optimización de una prueba de amplificación isotérmica mediada por bucle (LAMP) para la detección de ADN del gen *ompA* de CT en orina.

**Métodos:**

Con el fin de reducir el coste de la prueba, se aplicaron algunas modificaciones, entre las que se incluyen el empleo de la polimerasa *Bsm* y una tinción de gel de ácido nucleico. La extracción de ADN crudo (método-1) se realizó mediante centrifugación de la orina a 14.000 *g* durante 30 minutos, calentando los sedimentos a 95 °C durante cinco minutos y centrifugándolo de nuevo a 17.000 *g* durante un minuto. Para aumentar su sensibilidad, se realizó una dilución de inhibidores de la orina con lavados con solución salina tamponada con fosfato (método-2). El ensayo LAMP-SYBR GOLD se incubó a 56 °C durante 60 minutos, observándose cambios de color en la tinción de gel de ácido nucleico bajo luz ultravioleta. Se analizó la orina de 326 pacientes con enfermedades de transmisión sexual con LAMP-SYBR GOLD y con PCR en tiempo real, con el fin de comparar su sensibilidad y especifidad.

**Resultados:**

La sensibilidad analítica del ensayo LAMP-SYBR GOLD fue de 0,8 copias por volumen de reacción. Comparado con la PCR en tiempo real, el ensayo LAMP-SYBR GOLD demostró una sensibilidad del 71,4 %, una especifidad del 99,7 %, un valor predictivo positivo del 96,2 %, y un valor predictivo negativo del 96,7 %. Cinco de los siete falsos negativos obtenidos con el método-1 se volvieron a analizar con el método-2, obteniéndose el esperado resultado positivo en todos los casos un resultado positivo en todos los casos.

**Conclusiones:**

El ensayo LAMP-SYBR GOLD mostró una sensibilidad del 71 % y una elevada especifidad a la hora de detectar CT en orina mediante el método de extracción-1. La sensibilidad podría haber aumentado si se hubiera empleado el método-2, probablemente debido a la eliminación de inhibidores de la orina.

## Introducción

La *Chlamydia trachomatis* (CT) es el agente etiológico más común en la uretritis no gonocócica (UNG), con una prevalencia del 2,9 % en todo el mundo [1]. Las infecciones genitourinarias por CT suelen ser asintomáticas, causando complicaciones tales como enfermedad inflamatoria pélvica, embarazo ectópico e infertilidad por factor tubárico en las mujeres y epididimitis y proctitis en los hombres. Tanto si cursa de forma sintomática como asintomática, la infección se puede transmitir sexualmente [1]. En 2020, se comunicaron aproximadamente 129 millones de nuevos casos de infección genital por CT en todo el mundo [2]. En Sri Lanka, el porcentaje de CT en mujeres atendidas en las clínicas del Programa de Control de Enfermedades de Transmisión Sexual y SIDA (NASCP, por sus siglas en inglés) se incrementó del 8,3 % en 2012 [3] al 17,1 % en 2015 [4].

Las pruebas de amplificación de ácido nucleico (NAATs, por sus siglas en inglés), empleadas habitualmente en la clínica, son las que poseen mayor sensibilidad y especifidad para la detección de CT, considerándose el patrón oro. Aunque existen en el mercado NAAT aprobadas por la Food and Drug Administration (FDA) para el diagnóstico de infección por CT [5], su coste resulta muy elevado para un país de recursos limitados como Sri Lanka. El NASCP de Sri Lanka es el responsable de coordinar la respuesta nacional al VIH y a las enfermedades de transmisión sexual, en colaboración con distintos agentes nacionales e internacionales. El único laboratorio que realiza pruebas diagnósticas para la CT se encuentra en el NSACP. Debido a su elevado coste, las PCR en tiempo real únicamente se realizan a los pacientes sintomáticos, así como a individuos asintomáticos de alto riesgo. Es perentorio que en Sri Lanka se disponga de pruebas menos costosas, con una sensibilidad y especifidad adecuadas, para el diagnóstico de la infección por CT. La amplificacion isotérmica mediada por bucle (LAMP) es una nueva NAAT con elevada sensibilidad y especifidad.

Choopara y col (2017) publicaron un método LAMP para la detección de ADN de la proteína A de membrana externa *(ompA DNA)* en frotis endocervicales con una sensibilidad del 91 % y una especifidad del 95 % [6]. El genoma circular de CT consiste en un cromosoma único que contiene el gen *ompA*, que tiene una longitud de 1,2 kb. Dicho gen codifica para las principales proteínas de membrana externa que se emplean para clasificar la CT en 19 genotipos [7]. Para detectar el gen *ompA*, Choopara y col (2017) diseñaron cebadores LAMP dirigidos a seis regiones conservadas independientes [6]. Es más sencillo recoger muestras de orina que frotis endocervicacles y, además, son aplicables a ambos sexos. Nuestro objetivo era optimizar un método LAMP empleando cebadores previamente desarrollados [6] para detectar ADN del gen *ompA* de CT en orina.

## Materiales y métodos

### Recogida de muestras

Se recogió la primera orina de la mañana de los y las pacientes que acudieron a consulta en las clínicas del NSACP (n=126) y en el Hospital Colombo South Teaching de Kalubowila (Sri Lanka) (n=200) entre el 19 de marzo de 2018 y el 28 de octubre de 2019 (total n=326). Los criterios de inclusión fueron: edad ≥18 años, síntomas de infección urogenital (p.ej. secreción uretral, cervical, o vaginal anómala, elevación de leucocitos en las secreciones, coinfección por *Neisseria gonorrhoeae*) y pacientes asintomáticos con factores de riesgo (múltiples compañeros sexuales, hombres que practican sexo con hombres, trabajadoras del sexo, clientes de trabajadoras del sexo y personas con contacto con pacientes con cervicitis o uretritis). Se excluyó a los pacientes que hubieran orinado en las dos horas previas al reclutamiento y que hubieran estado tomando antibióticos en las tres últimas semanas. Para el cálculo de la tasa de prevalencia de infección por CT sintomática del 13,9 % y asintomática del 9,2 % [4], se calculó un tamaño muestral de 184 pacientes sintomáticos y 129 asintomáticos, con un margen de error de 0,05 y un intervalo de confianza del 95 % (fórmula estadística http://www.calculator.net/sample-size-calculator.html). Con anterioridad al examen físico de los pacientes, se recogieron 30 mL de la primera orina de la mañana, que se almacenó a 2–8 °C durante un máximo de siete días, para su posterior procesamiento. Se almacenaron alícuotas de orina fresca a −80 °C, con el fin de repetir el análisis durante el proceso de optimización.

### Orina de control positivo y negativo

Como control positivo, se utilizó una muestra de orina con resultado positivo tanto mediante PCR anidada [9] como por PCR en tiempo real Artus^®^ C. trachomatis Plus RG (Qiagen, Alemania), como control negativo, se utilizó una muestra de orina negativa en las dos pruebas. Los controles positivo y negativo se dividieron en partes alícuotas y se almacenaron a −20 °C para su uso en todas las pruebas.

### Extracción de ADN crudo para la prueba LAMP-SYBR GOLD (método-1)

La extracción de ADN crudo para la prueba LAMP-SYBR GOLD se realizó siguiendo el método descrito por Choopara y col, con algunas modificaciones (método-1) [6]. Se centrifugó la orina (1.5 mL) a 14.000 *g* durante 30 minutos. Se descartó el sobrenadante y se calentaron 40 µL del sedimento con 20 µL de *buffer* Tris-HCl 10  mM y EDTA 1 mM (pH 7,5) a 95 °C durante 5 minutos, empleando un Termobloque [6]. Se enfrió con hielo hasta que se aclaró la superficie del líquido. A continuación, se centrifugó a 17.000 *g* durante un minuto, empleando el sobrenadante para la prueba LAMP-SYBR GOLD. Los extractos de ADN crudos se prepararon el mismo día en que se realizó la prueba.

### Extracción de ADN crudo para la prueba LAMP-SYBR GOLD (método-2)

Modificamos el método-1 para mejorar aún más la sensibilidad de la prueba LAMP-SYBR GOLD. Las alícuotas (1,5 mL) de orina almacenadas, se descongelaron rápidamente en un baño de agua a 37 °C, y se centrifugaron a 14.000 *g* durante 30 minutos a temperatura ambiente. Se descartó el sobrenadante y se añadieron 200 µL de solución salina tamponada con fosfato (PBS; pH 7,4) para diluir los inhibidores urinarios. Seguidamente, se centrifugó a 14.000 *g* durante 30 minutos a temperatura ambiente. Tras eliminar el sobrenadante, se disolvió el sedimento en 200 µL de PBS y se calentó a 95 °C durante 5 minutos con 100 µL de *buffer* Tris-EDTA (pH 7,4). Se enfriaron los tubos en hielo y se centrifugaron a 17.000 *g* durante 1 minuto. A continuación, se recogió el sobrenadante para realizar la prueba LAMP-SYBR GOLD.

### Extracción de ADN de la orina con los kits QIAamp viral RNA

Este kit está recomendado para extraer ADN de bacterias en la orina [8]. La extracción se realizó siguiendo las instrucciones del fabricante para el protocolo de extracción. Se lisaron 140 µL de orina, añadiéndolos posteriormente a las columnas de *spin,* con *buffer* y centrifugándolos. Se eluyó el ADN en dicho tampón de elución. Finalmente, se almacenaron las alícuotas de ADN a −20 °C para repetir los ensayos.

### PCR en tiempo real

Se prepararon plantillas de ADN de la orina de los pacientes con el mini kit QIAmp viral RNA (Qiagen, Alemania). La PCR en tiempo real Artus^®^ C. trachomatis Plus RG (Qiagen, Alemania) se realizó siguiendo las instrucciones del fabricante y usando el control interno suministrado. Los resultados de la PCR en tiempo real se emplearon como referencia para determinar la sensibilidad y especifidad de la prueba LAMP-SYBR GOLD.

### PCR anidada

Utilizamos la PCR anidada descrita por Somani y col (2000) para confirmar los resultados de la PCR en tiempo real de los controles positivo y negativo [9]. Seguidamente, empleamos el ADN extraído con el mini kit QIAamp Viral RNA para realizar PCR anidada siguiendo el método descrito por Somani y col (2000), dirigido específicamente al gen *ompA* de CT [9].

### La prueba LAMP-Sybr Gold

Utilizamos los controles positivo y negativo para optimizar la prueba LAMP-Sybr Gold. Para la prueba LAMP-SYBR GOLD, se empleó un conjunto de seis cebadores publicados [6], esto es, FIP, BIP, F3, B3, FLP, BLP (Integrated DNA Technologies, EE. UU), dirigidos selectivamente al gen *ompA* de CT [6]. Se calcularon las condiciones óptimas para la prueba LAMP-SYBR GOLD para los componentes a continuación descritos: betaína (0,5 M, 0,8 M), MgSO_4_ (6–10 mM), ADN polimerasa *Bsm* (4U, 6U, 8U), volumen de plantilla (5–9 µL), temperatura de incubación (55–60 °C) tiempo de reacción (40 and 60 min), mediante electroforesis en gel para la detección.

Las condiciones optimizadas del ensayo LAMP-SYBR GOLD para 25 µL de reacción LAMP fueron: 7 µL de extracto crudo (método-1) en *buffer* 1× (20mMTris-HCl [pH 8.8], 10 mM de KCl (NH_4_)_2_SO_4_, 2 mM de MgSO_4_, 0,% (v/v) Tween 20), 1,6 µM de cebadores FIP y BIP, 0,2 µM de cebadores F3 y B3, 1,4 µM de cebadores FLP y BLP [10], con 1,4 mM de desoxinucleósido trifosfato (Qiagen, Germany) cada uno, betaína 0,8 M (Sigma Aldrich, EE.UU), 6 mM de MgSO_4_, 8U de polimerasa *Bsm* fragmento grande (Thermo Scientific, EE.UU). Previamente a la adición del extracto crudo, se depositó una capa de 12,5 µL de parafina líquida sobre la mezcla maestra, depositando la plantilla bajo dicha capa. Se suspendió un microlitro de gel de tinción de ácido nucleico Sybr^™^ Gold 10× (Invitrogen, EE. UU.; Cat. S11494) del interior de la tapa del tubo de reacción. Posteriormente, se incubó a 56 °C durante 60 minutos, deteniendo la reacción calentándolo a 80 °C durante 2 minutos. Una vez finalizada la incubación, la tinción Sybr^™^ Gold (Invitrogen, EEUU; Cat.No S11494) se incorporó a la mezcla de reacción mediante agitación vorticial. Las reacciones positivas se detectaron mediante fluorescencia amarilla intensa bajo luz UV de 320 nm.

### Sensibilidad y especifidad analítica

El ADN de CT extraído con el kit QIAmp viral RNA (Qiagen, Alemania) se cuantificó con un espectofotómetro NanoDrop^®^. La sensibilidad analítica del ensayo LAMP-Sybr Gold optimizado se determinó empleando orina de control negativo, enriquecida con 10 diluciones seriadas de ADN de CT cuantificado (7,49 ng–7,49 × 10^−4^ fg; 8,06 × 10^9^ – 8,06 × 10^−1^ copias). El producto del ensayo LAMP-SYBR GOLD se visualizó con luz UV de 320 nm y electroforesis en gel al 1,5 % con tinción de Sybr^™^ Gold (Invitrogen, USA; Cat. Nº S11494). La especifidad de los cebadores se confirmó realizando el ensayo LAMP-Sybr Gold con orina de control negativo enriquecida con ADN de virus del herpes simple tipo 2 *Neisseria gonorrhoeae*, *Trichomonas vaginalis, Candida albicans* (suministrado por el NSACP de Sri Lanka) y *Mycoplasma genitalium.*

### Sensibilidad y especifidad diagnóstica

La evaluación del ensayo LAMP-Sybr Gold con extracción de ADN crudo (método-1) se realizó empleando 326 muestras de la primera orina de la mañana procedentes de 189 pacientes sintomáticos y 137 pacientes asintomáticos de alto riesgo. El investigador que realizó el ensayo LAMP-SYBR GOLD desconocía los resultados de la prueba de PCR en tiempo real, que se empleó como prueba de referencia. Los resultados se obtuvieron mediante visualización del cambio de color con la lámpara UV. Los resultados del ensayo LAMP-SYBR GOLD discordantes, fueron repetidos.

## Resultados

### Optimización del ensayo LAMP-SYBR GOLD

Para la optimización inicial, se probaron diferentes métodos de extracción de ADN. El ADN extraído mediante hidróxido de sodio proporcionó un límite inferior de detección. El ADN extraído mediante calentamiento y precipitación con alcohol mostró un bajo nivel de pureza (datos no mostrados). Dado que el método de extracción de ADN crudo descrito por Choopara y col fue el que arrojó mejores resultados, se utilizó el método-1 para la optimización del ensayo LAMP-SYBR GOLD [6]. La optimización de la concentración de MgSO_4_ (véase la Figura 1 del Material Suplementario) reveló que el patrón de escalera más distintivo se producía a una concentración de 8 mM. La temperatura óptima de incubación (véase la Figura 2A del Material Suplementario) fue de 56 °C, mientras que, en la Figura Suplementaria 2B se observa la banda más clara con plantilla de 7 µL tras 60 minutos. La estimación del límite de detección (LOD, por sus siglas en inglés) a diferentes concentraciones de polimerasa Bsm (Figura 3 del Material Suplementario) y los distintos volúmenes de plantilla (Figura 4 del Material Suplementario) señalaron 8U de polimerasa Bsm y 7 µL de plantilla como las condiciones óptimas. Cada prueba de optimización se repitió tres veces. Con este ensayo LAMP, se evaluaron los indicadores de color visuales, esto es, azul de hidroxinaftol y bromuro de etidio, aunque resultó difícil distinguir el cambio de color en los puntos finales. De este modo, empleamos la tinción de gel de ácido nucleico Sybr^™^ Gold (Invitrogen, EE.UU; Cat.No.S11494) con luz UV. La tinción Sybr^™^ Gold emite una fluorescencia amarilla bajo la luz UV cuando se une a ADN (Figura 5 del Material Suplementario).

### Límite de detección (LOD)

Cuando se realizó la extracción de ADN crudo de la orina siguiendo rigurosamente el método descrito por Choopara y col, el LOD obtenido fue de 74,9 fg (8,06 × 10^4^ copias) mediante electroforesis en gel [6]. El ensayo LAMP de aproximación solo incrementó el LOD 10 veces (datos no mostrados). Con el fin de aumentar aún más el LOD, se centrifugaron las muestras de orina antes y después de su calentamiento (método-1) y se añadió la tinción de gel de ácido nucleico Sybr^™^ Gold (Invitrogen, EE. UU; Cat. Nº S11494) como indicador visual. Con las modificaciones realizadas sobre el método descrito por Choopara y col, se logró mejorar el LOD de los 74,9 fg iniciales a 7,49 × 10^−4^ fg, o aproximadamente 0,8 copias, si se visualizaba bajo luz UV. Se obtuvo el mismo LOD con la visualización con luz UV que con la electroforesis en gel. De este modo, al analizar las muestras clínicas, se empleó la visualización con luz UV para detectar el amplicón.

### Sensibilidad y especifidad diagnóstica

Al analizar las 326 muestras de orina con el ensayo LAMP-SYBR GOLD y con extracción de ADN crudo (método-1), se detectaron un falso positivo y siete falsos negativos. Se empleó la PCR en tiempo real como prueba de referencia (Tabla 1). Este ensayo mostró una sensibilidad del 71,4 % (20/28), una especifidad del 99,7 % (297/298), un valor predictivo positivo del 96,2 %, un valor predictivo negativo del 96,7 % y una precisión del 97,2 % (Tabla 1).

**Tabla 1:** Resultados de las 326 muestras de orina analizadas mediante extracción en crudo LAMP-Sybr Gold (método-1) y PCR en tiempo real.

	PCR en tiempo real positiva	PCR en tiempo real negativa	Total
LAMP con extracción en crudo (método-1) positivo	20	1	21
LAMP con extracción en crudo (método-1) negativo	8	297	305
Total	28	298	326

Seis de los siete falsos negativos y el falso positivo se observaron en pacientes sintomáticos. La sensibilidad y especifidad del ensayo LAMP Sybr-Gold fue similar en los pacientes sintomáticos y no sintomáticos (Tabla 2). El ensayo LAMP-SYBR GOLD no mostró reactividad cruzada con otros patógenos genitales, incluyendo el virus de herpes simple tipo 2, *N. gonorrhoeae, M. genitalium, T. vaginalis*, y *C albicans*, tal como indica la ausencia de patrones en escalera en el gel (véase la Figura 6 del Material Suplementario). Cinco de las siete muestras donde se obtuvieron falsos negativos se volvieron a analizar con el método-2 de extracción de ADN crudo. Las cinco muestras se visualizaron levemente positivas bajo luz UV, y la positividad se confirmó con el patrón en escalera observado en la electroforesis en gel (véase Figura 7 del Material Suplementario).

**Tabla 2:** Muestras de orina de pacientes sintomáticos y asintomáticos analizadas mediante PCR en tiempo real y LAMP-SYBR GOLD con extracción en crudo, método-1.

	PCR en tiempo real		
	Positivo	Negativo	Total
**Pacientes sintomáticos (n=189)**			

LAMP + extracción en crudo (método-1) Positivo	17	1	18
LAMP + extracción en crudo (método-1) Negativo	7	164	171
Total	24	165	189
Sensibilidad 70,8 %			
Especificidad 99,4 %			

Pacientes asintomáticos (n=137)			
LAMP + extracción en crudo (método-1) Positivo	3	0	3
LAMP + extracción en crudo (método-1) Negativo	1	133	134
Total	4	133	137
Sensibilidad 75 %			
Especificidad 100 %			

## Discusión

Choopara y col (2017) desarrollaron un ensayo LAMP de elevada sensibilidad (91 %) y especifidad (95 %) para detectar CT en frotis endocervicales [6]. Dado que la recogida de muestras de orina es más sencilla, menos invasiva y se puede realizar en ambos sexos, realizamos algunas modificaciones del método de Choopara y col (2017) para poder detectar CT en la orina. Mediante el empleo de cebadores previamente publicados [10], desarrollamos una prueba LAMP-SYBR GOLD para orina. Nuestra LAMP-SYBR GOLD mostró una sensibilidad del 71,4 %, una especifidad del 99,7 % y un LOD de 0,8 copias de ADN de *ompA* de CT por cada reacción, siendo la tinción Sybr^™^ Gold el indicador visual utilizado (véase la Figura 6 del Material Suplementario).

Una de las modificaciones realizadas del protocolo desarrollado por Choopara y col fue el empleo de la polimerasa *Bsm* [6]. El coste de l*a* polimerasa *Bsm* es un tercio del precio de la polimerasa *Bst*, y es más accesible en Sri Lanka. Con el fin de reducir aún más los costes, los cebadores de bucle no se purificaron mediante HPLC. Dichas modificaciones podrán ser las causantes de que el periodo de incubación se prolongara 15 minutos más. Al aplicar a la orina el protocolo de Choopara y col (2017) con las modificaciones anteriormente descritas, el LOD fue de 8,06 × 10^4^ copias de ADN de *ompA* de CT, frente al LOD de 1,8 × 10^2^ copias que se obtuvo con el protocolo original para las muestras endocervicales [6]. Se empleó la prueba LAMP *touchdown* de aproximación para intentar aumentar la sensibilidad analítica, pero solo logramos aumentar el LOD diez veces (datos no mostrados). Se realizaron algunas modificaciones del método de extracción descrito por Choopara y col (método-1): 1) se centrifugó la orina a 14.000 *g* durante 30 minutos y se recogió el sedimento para la extracción por calor, 2) tras la extracción por calor, se centrifugó a 17.000 *g* durante un minuto y se recogió el sobrenadante para el análisis mediante LAMP-SYBR GOLD. Esta modificación de la extracción de ADN crudo aumentó el LOD significativamente. La tinción de gel de ácido nucleico Sybr^™^ Gold (Invitrogen, EE.UU; Cat.No.S11494) es entre 25 y 100 veces más sensible que el bromuro de etidio y puede ser utilizada a una dilucion alta. La modificación del método de extracción, la elevada sensibilidad de la tinción Sybr^™^ Gold (Invitrogen, EE.UU; Cat. Nº S11494), y el elevado volumen de plantilla (7 µL), contribuyeron a aumentar el LOD a 8,06 x10^−1^ (0,8) copias. Se evitó la contaminación del ADN colocando una capa de parafina sobre la mezcla maestra y depositando la plantilla debajo de la capa de cera.

Los factores inhibidores de la orina en la plantilla de ADN genómico crudo podrían explicar la baja sensibilidad clínica, a pesar del LOD de 0,8 copias de ADN de *ompA* de CT por cada reacción. En segundo lugar, la cantidad de ADN genómico liberado calentando 40 µL de sedimento urinario podría haber sido insuficiente. Es necesario optimizar el volumen de sedimento urinario para la extracción por calor. Los inhibidores urinarios son los responsables de la reducida eficacia de los ensayos LAMP en orina. Edward y col (2014) determinaron que las reacciones LAMP podrían resistir mayores niveles de urea que los hallados en la orina humana. Jevtuševskaja y col (2016) establecieron que la albúmina sérica bovina, Mg^2+^ y la urea a cantidades fisiológicas no afectan al proceso LAMP [11], [12]. En nuestra prueba de sensibilidad analítica, la orina negativa para CT se enriqueció con ADN de CT purificado. Por lo tanto, es improbable que la baja sensibilidad clínica observada en nuestro ensayo LAMP-SYBR GOLD se deba a la urea.

En el método-2 para extracto crudo, con el fin de eliminar la presencia de posibles inhibidores de la orina, se centrifugaron los sedimentos de la orina con PBS (paso de lavado). Este método se aplicó a cinco muestras de pacientes que fueron positivas en la PCR en tiempo real, pero negativas con el método-1 de extracción de ADN crudo. Los resultados vagamente positivos obtenidos en las cinco muestras indican que la eliminación de los inhibidores de orina mediante el método-2 podría mejorar la sensibilidad del ensayo LAMP-SYBR GOLD (Figura 9 del Material Suplementario). Siguiendo la optimización de la cantidad del sedimento urinario, es necesario volver a analizar todas las muestras de los pacientes con el método-2 de extracción para determinar la verdadera sensibilidad clínica de este ensayo LAMP-SYBR GOLD. El reto a la hora de desarrollar pruebas diagnósticas para los países de recursos limitados es lograr un equilibrio entre el coste y la eficacia de la prueba. Aunque los kits de extracción comerciales mejoran la sensibilidad de las pruebas LAMP, su elevado coste los hace inasumibles para los países pobres. Tal como demuestran diferentes estudios, la extracción de ADN crudo por calentamiento es una forma rápida, económica y adecuada para los ensayos CT LAMP [6], [12].

Desde la introducción del método LAMP por Notomi y col (2000), se han desarrollado escasas pruebas CT LAMP, y las que han surgido, son para muestras endocervicales [6], [8], [13], [14], [15], [16]. La única prueba CT LAMP para orina, desarrollada por Jevtuševskaja y col (2016), precisa tratamiento previo con una mezcla de lisis de péptidos antimicrobianos, mostrando una sensibilidad del 73 % y una especificidad del 100 %, con un LOD de 25 copias de plásmidos por reacción [12]. Con la disponibilidad de kits comerciales de PCR en tiempo real para CT de elevada eficacia, ha disminuido el interés por desarrollar otros métodos diagnósticos más económicos para los países de recursos limitados. Nuestro ensayo LAMP-SYBR GOLD para orina resultó ser sensible y altamente específico para la detección de ADN del gen *ompA* de CT en orina, empleando la tinción Sybr^™^ Gold como indicador visual. Se trata de un método económico (el precio de los reactivos para un solo espécimen es de aproximadamente un dólar americano), sencillo y más adecuado para los países de recursos limitados. La realización del proceso en su totalidad requiere entre una hora y una hora y media. Los resultados obtenidos demuestran que el ensayo CT LAMP-SYBR GOLD en orina es un método coste-efectivo, que se puede seguir mejorando. Optimizamos y validamos clínicamente el ensayo CT LAMP-SYBR GOLD utilizando el método-1 de extracción. Sin embargo, no logramos los mismos resultados con el método-2 de extracción. Aunque el método-2 es prometedor, su eficacia aún requiere validación mediante estudios independientes. Para los países que no se pueden permitir el elevado coste de PCR en tiempo real de gran eficacia, este método CT LAMP-SYBR GOLD se postula como una alternativa menos costosa para el diagnóstico de la infección por CT.

## Supplementary Material

Supplementary Material

## Material Suplementario

Este artículo incluye material suplementario (https://doi.org/10.1515/almed-2025-0061).
