# Supplementary material for: Detección de ADN del gen ompA de Chlamydia trachomatis en orina mediante amplificación isotérmica mediada por bucle (LAMP)
Source: Adv Lab Med. 2025 May 29;6(3):320–6. [Article in Spanish] doi: 10.1515/almed-2025-0061 (PMC12446913; doi:10.1515/almed-2025-0061)
Supplement: Supplementary file 1 — Supplementary Material [file j_almed-2025-0061_suppl_001.docx]

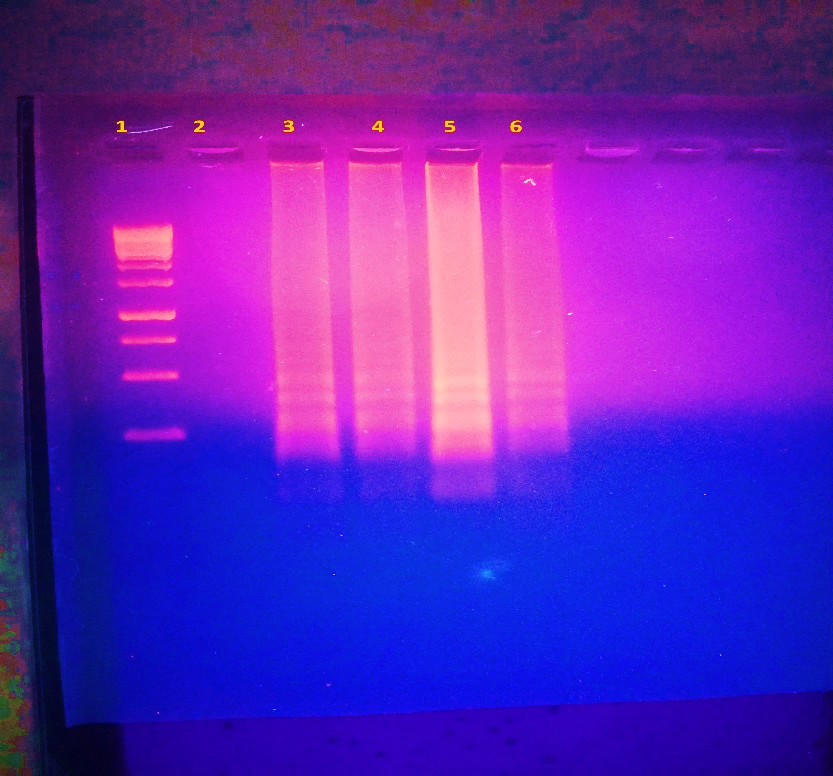


**Material suplementario - Figura 1.** Optimización de la concentración de MgSO_4_ mediante electrophoresis en gel como método de detección. Banda 1, escalera de ADN de 1 kb; banda 2, vacía; banda 3, 6mM de MgSO_4_; banda 4, 7mM de MgSO_4_; banda 5, 8mM de MgSO_4_ (**óptima**); banda 6, 10mM de MgSO_4_; banda 7, control negativo con 8mM de MgSO_4_.


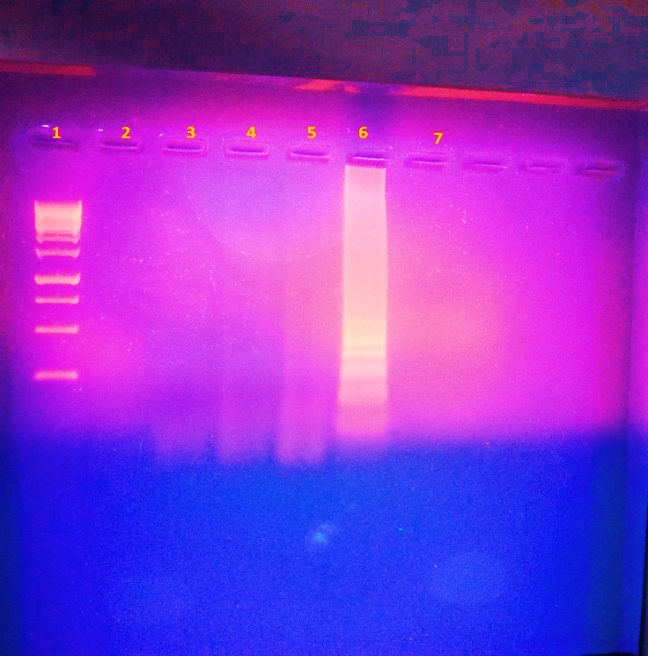

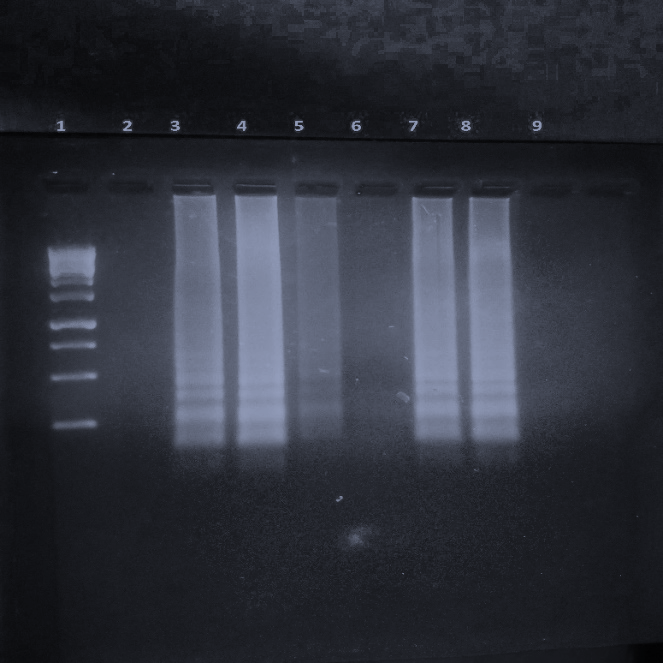


A

B

**Material suplementario - Figura 2.** (A) Optimización de LAMP para la temperatura de incubación (55^o^C-60^o^C). Banda 1, escalera de ADN de 1 kb; banda 2, vacía; banda 3, 55^o^C; banda 4, 56^o^C (óptimo); banda 5, 57^o^C; banda 6, 58^o^C; banda 7, 59^o^C; banda 8, 60^o^C; banda 9, control negativo a 56^o^C. (B) Optimización de la duración de la incubación (40 y 60 minutos) a 56^o^C con 5-7 µl de plantilla a diferentes concentraciones. Banda 1, escalera de ADN de 1 kb; banda 2, vacía; banda 3, 5µl de una dilución 10^-2^ del control positivo (CP) incubado durante 40 minutos; banda 4, 6µl de dilución 10^-2^ de CP incubado durante 40 minutos; banda 5, 7µl de una dilución 10^-2^ de CP incubado durante 40 minutos; banda 6, 7µl de dilución 10^-3^ de CP incubado durante 60 minutos (**óptimo**); banda 7, 7µl de control negativo incubado durante 60 minutos.


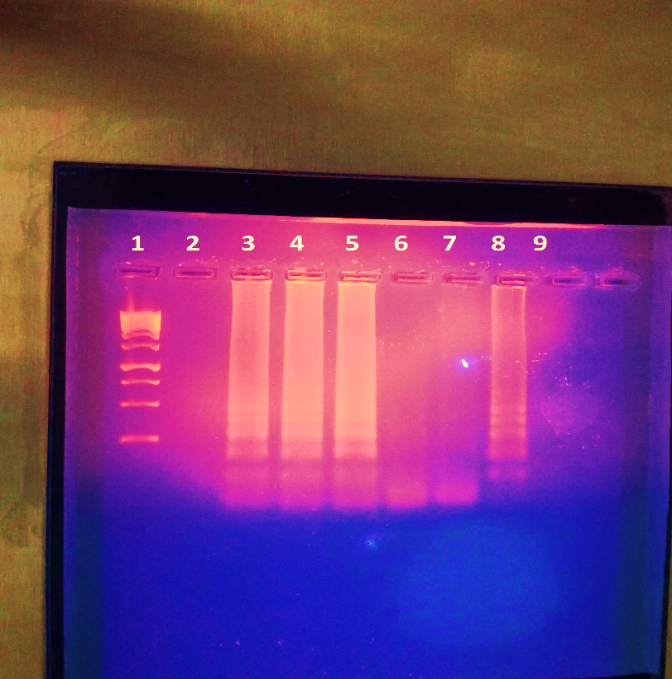

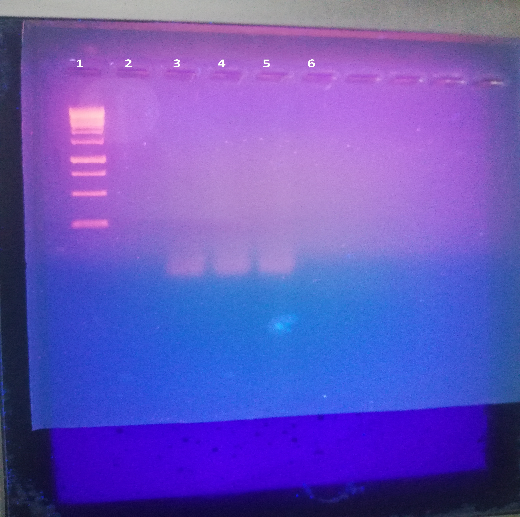


A

B

**Material suplementario - Figura 3**. (A) Optimización de LAMP para polimerasa *Bsm* (4-6U). Banda 1, escalera de ADN de 1kb; banda 2, vacía; bandas 3-5, 4U, 6U, 8U de enzima con un el control positivo diluido a 10^-1^; banda 6-8, 4U, 6U, 8U (**óptima**) de enzima con un control positivo diluido a 10^-2^; banda 9, control negativo con 8U de enzima. (B) Banda 1, escalera de ADN de 1kb; banda 2, vacía; bandas 3-5, 4U, 6U, 8U de enzima con el control positivo diluido a 10^-3^; banda 9, control negativo con 8U de enzima.


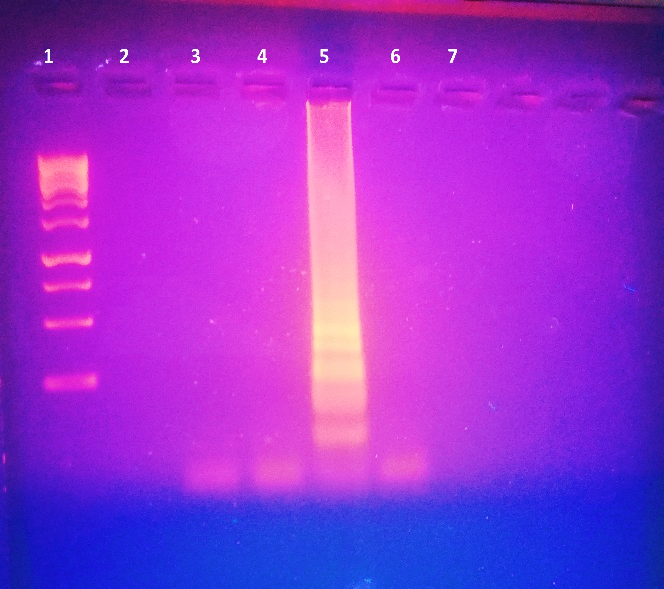


B

**
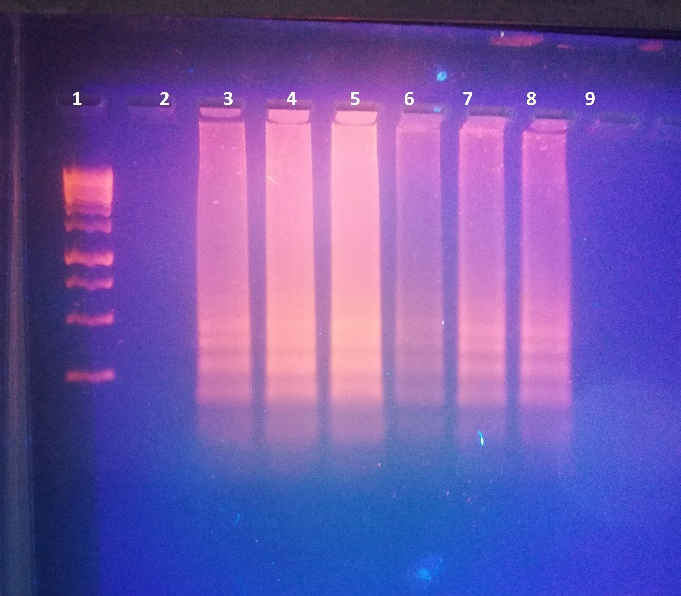
Material suplementario- Figura 4**. (A) Optimización de LAMP para el volumen de plantilla. Banda 1, escalera de ADN de 1kb; banda 2, vacía; bandas 3-5, 5µl, 6µl, 7 µl de diluciones a 10^-1^ del control positivo; bandas 6-8, 5µl, 6µl, 7 µl de diluciones al 10^-2^ del control positivo; banda 9, 5µl de control negativo sin diluir. (B) Banda 1, escalera de ADN de 1 kb; banda 2, vacía; bandas 3-5, 5µl, 6µl, 7µl **(optimum)** de diluciones al 10^-3^ de control positivo; banda 6, 7µl de diluciones al 10^-4^ de control positivo; banda 7, 7µl de control negativo no diluido.

A

BB

A


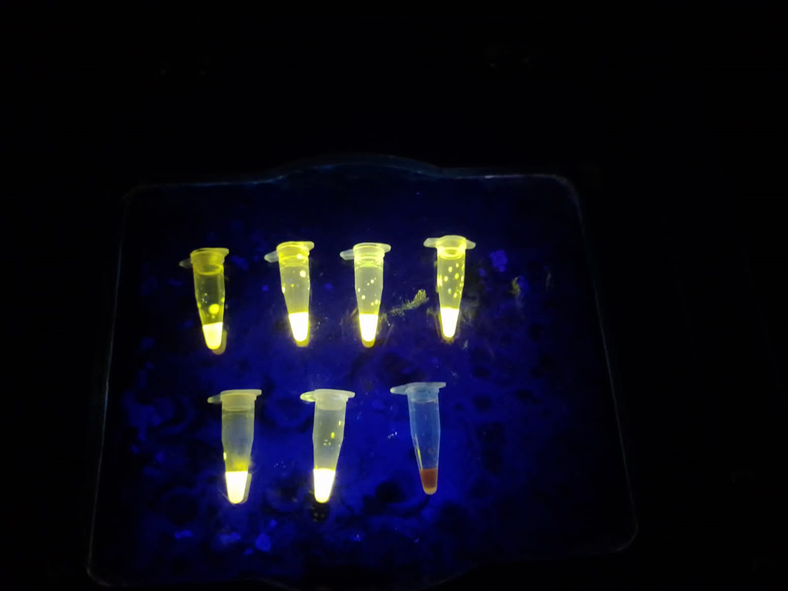


**Material suplementario- Figura 5**. Controles positivo (A) y negativo (B) de LAMP con tinción de ácido nucleico Sybr™ Gold observados bajo luz ultravioleta.


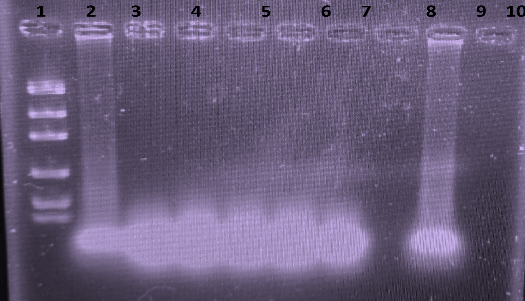


10^-10^  10^-11^ 10^-12^

**Material suplementario- Figura 6**. La especificidad de los cebadores se confirma mediante orina enriquecida con AND del virus de herpes simple tipo 2 (banda 3), *Neisseria gonorrhoeae* (banda 4), *Mycoplasma genitalium* (banda 5), *Trichomonas vaginalis* (banda 6), *Candida albicans* (banda 7), vacía (banda 8), y el control positivo (bandas 2 y 9).


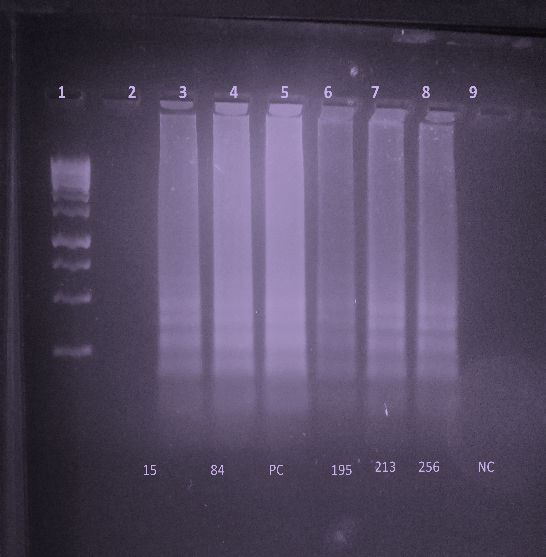


**Material suplementario- Figura 7**. Los cinco especímenes que dieron un falso negativo (especímenes número 15, 84, 195, 213 y 256) se volvieron levemente positivos (patrón en escalera) cuando se utilizó el método-2 de extracción. Aumentar el volumen de orina para la extracción y dilución de los inhibidores de orina fueron pasos adicionales empleados en el método-2.
